# The Impossibility of a Moral Right to Privacy

**DOI:** 10.1007/s12152-022-09500-3

**Published:** 2022-06-28

**Authors:** Ingmar Persson, Julian Savulescu

**Affiliations:** 1 Göteborg University, Gothenburg, Sweden; 2 The Oxford Uehiro Centre for Practical Ethics, University of Oxford, Suite 8, Littlegate House, St Ebbe’s Street, Oxford, OX1 1PT UK; 3 Murdoch Childrens Research Institute, Melbourne, Australia; 4 University of Melbourne, Melbourne, Australia

**Keywords:** Right to privacy, Information acquisition, Means of information acquisition, Uses of information, Omniscience

## Abstract

This paper clarifies and defends against criticism our argument in *Unfit for the Future* that there is no moral right to privacy. A right to privacy is conceived as a right that others do not acquire information about us that we reserve for ourselves and selected others. Information acquisition itself is distinguished from the means used to acquire it and the uses to which the information is put. To acquire information is not an action; it is to be caused to be in an internal state. By contrast, means of acquisition and uses of information are actions that can be voluntarily controlled. We can therefore have rights against others that they stay away from certain means and uses but not from information acquisition in itself. An omniscient, omnipotent and omnibeneficient being is not thought to violate a right to privacy because its means and uses of information are morally acceptable.

## The Claim

In *Unfit for the Future* [1]*,* we argued that there is no moral right to privacy. This argument was criticized by Jan Christoph Bublitz in this journal[2] and defended by us in the same issue [3]. More recently, our argument has met opposition from Björn Lundgren [4]. So, our argument is in need of further defence and clarification. It is an argument that could be consistently accepted, though other moral rights are endorsed, such as rights to our bodies and private property; and we shall here assume, in accordance with common sense morality, that we have such rights.

To begin with, it should be noted that it is a *moral* and not a legal right to privacy that we are talking about. If we have a moral right to something, it is often the case that we morally should have a legal right to protect it – often but not always. For instance, you may have a moral obligation or duty – we treat these terms as synonymous – to allow yourself to be tortured to death if this is the only way in which a million of innocent people can be saved from being similarly tortured to death. Then these people have a corresponding moral right against you to be saved by this means. But it is not true that there should be a law to this effect because the probability that anyone will ever find themselves in the situation of having to put up with this sacrifice is extremely low.

Many people believe that human beings have a moral right to privacy. A significant portion of those who have this belief are probably Christians who believe in God. But God, as conceived by Christians, is commonly thought to be an omniscient, omnipotent and omnibeneficent or morally perfect being. If we have rights to privacy, the conception of such a being would be *inconsistent*, for by being omniscient, it would violate the right to privacy of countless human (and non-human?) beings and, thus, would not be morally perfect. The divine being has acquired as much information about us as is conceivable; yet it has not violated our alleged right to privacy. How is that possible if we have such a right?

Our answer is that we do not have such a right, but this does not answer the question why people who believe that we have a right to privacy have failed to see that, if this is so, God must violate it. To this we reply that there are moral requirements closely related to the right to privacy that people regularly infringe but that a perfect being would never infringe. If the existence of these moral requirements is confused with the existence of a moral right to privacy, the fact that these requirements are necessarily respected by a perfect being could make people overlook their existence in this context while they are alert to their existence in the context of people who break them now and then. Thus, it is only in the latter context in which people who are guilty of the confusion indicated have occasion to attend to a moral right to privacy.

What are these closely related moral requirements? We take a right of people to privacy to be a right that outsiders do not acquire true beliefs or information about them that they reserve for themselves and a select group of others (2012: 53). The related requirements concern the *means* used to acquire information and the *uses* to which information is put (2012: 53; 2019: 35). These means and uses could violate the rights of people to their bodies and property and cause harm. The wrongful means used to acquire information could be invasive and transgress the boundaries of the bodies or property of people, and the information acquired may be used to blackmail or threaten people. But the right to privacy is about the acquisition of information in itself; if everything is there is to know about people is generally known, they clearly have no privacy, no private sphere inaccessible to the minds of others, however this extensive information is acquired. So, we think that a right that protects us merely against interference with our bodies and material property does not go far enough to qualify as a right to privacy. 

Being omnipotent, however, God has available all possible means of information-acquisition and being omnibeneficient will choose the best of them, and will put the information acquired to the best possible use. So, there is no need to appeal to the requirements about means and uses or a moral right to privacy with which they are conflated. No wonder, then, that it has not occurred to Christian believers in a right to privacy to accuse their God of innumerable violations, though they must be aware that this is an area in which they cannot keep any secrets.

Using means to acquire information and using the information acquired to attain certain ends are *actions* that could be performed intentionally or voluntarily. It takes actions or omissions to act to violate or respect rights by contravening or discharging the corresponding obligations. By contrast, to acquire information is in itself to be *caused* to be in a state and, to boot, a state *that is internal to the subject*. While acting is being a *cause* of something, acquiring information is being at the *effect*-end, being acted on or affected in some way. How could others have a moral right against subjects that they are not put in such an internal state that is not under the subjects’ voluntary control but an effect that they undergo? Otherwise expressed, how could subjects be under a moral obligation not to be caused to be in such an internal or private state? Put in a nutshell, this is the reason why we cannot see how acquiring information about someone else could in itself violate a right of privacy of them or fail to discharge an obligation to them.

It may be useful to compare acquisition of information with having an emotion. Having an emotion is also being passively affected – hence, the term ‘passion’ – and it is also primarily an internal state, though it often enough involves overt signs, like blushing and trembling. Emotions, like beliefs, are effects that are largely beyond our direct control. That is, we may avoid having emotions by avoiding situations in which we risk being exposed to them, but if we are in those situations, we cannot help feeling them to some extent. We can usually prevent ourselves from running away in fear or striking out in anger, but not feeling dryness of the throat or pounding of the heart in fear or some muscular tension or redness in the face in anger.

To the extent that we are able to control our emotional actions, we may be under an obligation to do so. Thus, others may have a right against us that we do not run away in fear and leave them in a fix or harm them by an angry blow. But we are under no obligation to have or to refrain from having emotions in so far as they are uncontrollable internal states of feeling certain bodily changes; consequently, others do not have rights to demand that we are or are not in these states.

It may be objected that there are emotions that a morally good or bad; for instance, it is morally good to feel compassion because someone suffers, and morally bad to feel *schadenfreude* because of their suffering – indeed, according to Schopenhauer, *schadenfreude* is the most ‘infallible sign of a thoroughly bad heart and profound moral worthlessness’.[5, p135]. Bu﻿t it is one thing to say that it is morally bad or immoral to feel emotions like *schadenfreude* and envy and another to say that there is a duty not to feel them. Even though we cannot prevent ourselves from having such emotions at one go, we may gradually rid ourselves of them. And we may have an ideal of completely discarding them and loving even our enemies, though we realize that we are unlikely to achieve this fully. But we do not have a *duty* to dispose of these emotions; this is not anything that we are morally *required* to do. It is something that goes beyond the call of duty; it is *supererogatory*.

Returning to the acquisition of information, we have suggested that it resembles emotions in being an internal state of being passively affected which is not directly controllable. For instance, we acquire information about our surroundings by the causal impact they have on our sense organs. If we have a functioning sense of sight and our eyes are open, we cannot help acquiring information about how things around us look. Certainly, we can prevent ourselves from acquiring this information by closing our eyes but that is a form of *indirect* control, control *by* doing some action. There are mental acts and operations like paying special attention to the information acquired or trying to suppress it, but the acquisition itself is not an act. Forming beliefs are not acts that we can perform at will; beliefs are designed to be determined by and fit the facts.

The fact that the rights of some correspond to the obligations of others to them indicates that this pair is applicable to *interpersonal* relations. Actions that affect others are such relations, and they can be voluntarily controlled. Acquiring information and experiencing emotions are states internal to subjects that cannot be (fully) voluntarily controlled. Therefore, the concepts of rights and obligations are not applicable to them.

In the case of acquisition of information as opposed to that of experiencing emotions, we cannot draw a distinction between information that it is *in itself* morally good or bad to acquire. For example, it is not in itself morally bad to acquire information about how someone looks naked, though it may be undesirable to the naked person. Nor is it in itself morally good to acquire information that enables us to save somebody from a lethal threat. In contrast to at least some emotions, the state of acquiring information is in itself evaluatively neutral.

## The Criticism

Let us now turn to our critics. Lundgren picks up and develops a counter-example first presented by Bublitz, an example of gawking at a person who lies naked and helpless in the street. Suppose that, when walking along street, you unexpectedly discover a person lying naked in front you. Lundgren agrees with us that ‘it is fair to say that by seeing that person nude (only for a moment), you have not violated that person’s right to privacy’ (2021: 112). So, we are in agreement with him that people do not have a moral right to privacy that is violated if we acquire private information about them by seeing them for a short period of time. We then argue that ‘it would be odd if such a right eventually kicks in if this period [of seeing] is gradually extended: how could the mere passage of time bring a *moral* right into existence?’ (2019: 36).

Lundgren objects: ‘It is clear that one consequence of extending the action temporally is that over time you would acquire *more* true beliefs about that person. Hence, on Persson and Savulescu’s definition, extending an action in time can violate a right to privacy’ (2021: 112; 116, 117–8). First, although it is often true that the longer you observe something, the greater the body of information you acquire about this object, this is not necessarily true. In the situation under consideration all the sensitive information may well be open to view so that you could collect it by a quick glance. But let us imagine that by prolonging your observation, you get a better look at the person’s body and acquire more information about it. It should be clear that, on our view, such an increase of the information acquired does not by itself bring you any closer to violating a right to privacy. For on our view what is decisive is not the *amount* of information acquired: it is the fact that acquiring information is not acting, or causing, but being caused to be in an (internal) state and, for this reason, cannot violate a right. It is the *nature* of the state of information acquisition that prevents it from being rights-violating; the amount of information is irrelevant. Remember, even the omniscience of God does not violate a right to privacy.

To prolong the observation of the naked person could however be an intentional action that is likely to be morally wrong. You are taking advantage of this person’s unfortunate circumstances to get a good look at his/her naked body. Instead, you should probably check whether this person is in need of urgent help; it is after all unlikely that somebody is lying naked in the street if they are perfectly all right. You may also cause this person to feel humiliated by your persistent stare if (s)he is conscious. In short, you are using morally unacceptable means to get better informed about the naked body of this person. Initially, when stumbled on this person, you acquired information about these matters by innocent means. Thus, a moral change from innocence to guilt occurs as your observation is prolonged, but it is a difference in the morality of the means employed to acquire information, not a difference consisting in that the information acquired grows until it violates a right to privacy. Lundgren is, then, quite mistaken when he concludes that our account implies that ‘you would not violate any right (to privacy or otherwise) of that person by *continuing* to look at her’ (2021: 115–6). Continuing to look is an act that may well violate a right; the increase of information likely to be gained by this means does not.

In a footnote, Lundgren suggests that we claim that ‘information acquisition is permissible because it is not “under the control of our will”’ (2021: 113, n3). But he thinks that this idea ‘fails’ for the example under discussion because information is here acquired by the voluntary action of prolonging the observation. However, this just goes to show that he overlooks the importance of our distinction between the state of acquiring information in itself and the means used to acquire it. Although the formation of beliefs in itself is not an act that can be executed voluntarily, true beliefs can be acquired by voluntary means that may be permissible rather than impermissible. For instance, continuing to look at the naked person may be permissible if it is done to find out whether (s)he is in need of assistance.

Lundgren regards the omniscience of a divine being as our ‘best example’ but he nevertheless demotes it to the humble place of a parenthesis in a footnote (2021: 113, n3). And his response to it is misdirected: it is that this being ‘cannot help knowing things about us’ (2021: 113, n3); its acquisition of information is not voluntarily conducted. But this being is supposedly omnipotent, so it should be able to curtail its omniscience should its goodness demand it, though it may not be able to form beliefs at will. However, we need not get embroiled in these theological speculations, since we maintain that information acquisition can be morally permissible regardless of whether or not the means of acquisition are voluntarily controlled. The thought-experiment of an omniscient being strikes us as being as close to conclusive as anything you might hope to find that there is no right to privacy. For if there had been such a right, it could not have failed to be maximally violated by a being who is omniscient.

In everyday circumstances, our privacy is protected by the rights we have to our bodies and property.^1^ But we argued in our book that this protection of privacy in our sense is only *contingent*. For instance, it is conceivable that some beings are equipped with super-senses that could penetrate this protection. Some beings might be mind-readers who by means of a passing glance into our eyes are capable of collecting all information we possess about ourselves; other beings might have X-ray vision for which both our bodies and lodgings are transparent. When these beings look into our eyes or at our lodgings, they would not affect them more than ordinary people do with their looks. Therefore, it could not be claimed that these god-like beings violate our rights to our bodies and property. It might be impossible to prevent them from gathering all the information about us they desire, unless we restrict their rights or liberty so harshly that it would be hard to justify it, for example, by putting them in prison or surgically removing their super-senses.

In our book (2012: 55), we went along with Judith Thomson’s rejection of ‘belief-mediated’ distress as a ground for holding that a right has been violated [6, 253–7]. For﻿ ﻿instance, if somebody believes it to be an intolerable insult if we do not compliment them all the time and is terribly distressed by our failure to do so, this does not suffice to show that they have a right to this effect. But even if this view is correct, the production of such distress could provide some moral reason to refrain from acting to acquire information, for rights and obligations are not all there is to common-sense morality. However, we shall not pursue the issue of how much protection of our privacy such reasons could supply. So, we do not offer any substantive guide to what could make means of information acquisition morally unacceptable or what uses of the information acquired are morally unacceptable. Our main claim is simply that a moral right to privacy as here conceived cannot possibly exist because of the nature of information acquisition.

## Footnotes

1 This should be kept in mind in view of Lundgren’s complaint that our ‘information-based’ account of the right to privacy does not cover ‘bodily, decisional or spatial privacy’ (2021: 115, n. 11). There are other rights that ensure such-like private spheres.
